# Draft Genome Sequence of a *Staphylococcus aureus* Isolate Taken from the Blood of a Preterm Neonatal Blood Sepsis Patient

**DOI:** 10.1128/genomeA.00906-14

**Published:** 2014-09-11

**Authors:** K. A. Kropp, A. Lucid, J. Carroll, V. Belgrudov, P. Walsh, B. Kelly, K. Templeton, C. Smith, P. Dickinson, A. O’Driscoll, P. Ghazal, R. D. Sleator

**Affiliations:** a Department of Biological Sciences, Cork Institute of Technology, Bishopstown, Cork, Ireland; b NSilico, Cork, Ireland; c Department of Computing, Cork Institute of Technology, Bishopstown, Cork, Ireland; d Division of Pathway Medicine, University of Edinburgh, Edinburgh, United Kingdom; e Microbiological Diagnostic Unit, Royal Infirmary, University of Edinburgh, Edinburgh, United Kingdom

## Abstract

Herein, we report the draft genome sequence of *Staphylococcus aureus* ED-NGS-1006, cultivated from a blood sample taken from a neonatal sepsis patient at the Royal Infirmary in Edinburgh, Scotland, United Kingdom.

## GENOME ANNOUNCEMENT

*Staphylococcus aureus* is a Gram-positive, clinically important pathogen, and it is one of the leading causes of blood sepsis (1–3). Preterm neonates are a highly susceptible patient group for bacterial infections (3–5), and rapid detection of blood sepsis and identification of the causative agent are critical to enable proper treatment (6–8). The ClouDx-i project aims to extend current knowledge of circulating pathogenic strains linked with blood sepsis in neonates to help inform the development of new and improved molecular diagnostics. Herein, we present the draft genome of a *Staphylococcus aureus* strain, isolated from a preterm neonate at the Royal Infirmary in Edinburgh in 2013. Positivity for blood sepsis and species were confirmed by classical microbiological identification and characterization techniques.

The isolate was grown overnight at 37°C on Luria broth (LB) agar, and genomic DNA was isolated using Qiagen genomic tips (Venlo, Limburg, Netherlands). Genomic DNA was fragmented (fragments 2 to 10 kb) using sonication and a non-size selected genome library was produced, using the Nextera mate pair kit (Illumina, San Diego, CA). This library was then sequenced on an Illumina MiSeq using MiSeq Reagent kit v3. Genomic sequence assembly, analysis, and automated reporting was carried out using Simplicity (9). This approach produced 2,971,922 total reads, resulting in an average 223-fold coverage. The average G+C content was 32.93%. For sequence assembly, a *de novo* assembly pipeline based on the Spades 3.10 assembly tool was used with k-mers K21, K33, K55, K77, K99, and K127 nucleotides in length, resulting in a total of 186 contigs, of which 11 were >1,000 bp representing 97.23% of total sequence information, with the largest contig being 1,056,944 bp in size. Post assembly processing was performed by Spades and only scaffolds >1,000 bp were considered when estimating genome length as 2,814,370 bp. We annotated the contigs with Prokka (10) and used the identified 16S rRNA gene to confirm the species as *Staphylococcus aureus*. A scaffold of the genome was produced with Contiguator2 and we identified the closest related strains by BLASTing the scaffold, returning strains *S. aureus* spp. *aureus* MRSA252 and Z172 as closely related but not identical isolates, evident by many small insertions and deletions in the genomes. The genome was then screened using Glimmer3 (11) identifying 2,629 ORFs. The predicted ORFs were compared to the Uniprot-Trembl database (12) using BLASTp, mapping 1,800 ORFs to the database. To identify potential virulence factors in the genome we further compared it using BLASTp to a local database built from the VFDB (13) and Victors databases. A 75% amino-acid sequence identity cut-off was used while only considering alignments longer than 100 amino-acids, identifying 84 hits.

Samples were handled in accordance with local ethical approval by the ethics committees of the NHS Lothian SAHSC Bioresource and NHS R&D office, Project ID: 2011/R/NE/01 and the HSS BioResource RequestID: 13/ES/0126.

### Nucleotide sequence accession numbers.

This whole-genome shotgun project has been deposited at DDBJ/EMBL/GenBank under the accession no. JPWO00000000. The version described in this paper is version JPWO01000000.
